# The Traditional Chinese Medicine Gedan Jiangya Decoction Alleviates Left Ventricular Hypertrophy via Suppressing the Ras/ERK1/2 Signaling Pathway

**DOI:** 10.1155/2022/6924197

**Published:** 2022-11-18

**Authors:** Shadi A. D. Mohammed, Hanxing Liu, Salem Baldi, Pingping Chen, Yu Wang, Fang Lu, Shumin Liu

**Affiliations:** ^1^Graduate School of Heilongjiang University of Chinese Medicine, Harbin 150040, Heilongjiang, China; ^2^Research Center of Molecular Diagnostics and Sequencing, Axbio Biotechnology (Shenzhen) Co., Ltd, Shenzhen, Guangdong 518057, China; ^3^Institute of Traditional Chinese Medicine, Heilongjiang University of Chinese Medicine, Harbin 150040, Heilongjiang, China

## Abstract

Gedan Jiangya Decoction (GJD), a Chinese herbal medicine composed of six botanical medicines, was designed to treat hypertension (patent published number (CN114246896A)). The overexpression of the ERK (extracellular signal-regulated kinase) signaling pathway is essential in developing left ventricular hypertrophy (LVH). This study aimed to evaluate GJD's effects on LVH in spontaneously hypertensive rats (SHRs) and examine its potential mechanisms on Ras/ERK1/2 pathway regulation. Thirty-five ten-week-old SHRs were randomly assigned to one of five groups: GJD low dosage, medium dose, high dose, model, and captopril. Wistar–Kyoto (WKY) rats served as the control group. All rats received a 6-week treatment. The following parameters were measured: systolic (SBP) and diastolic blood pressure (DBP), left ventricular mass index (LVMI), and serum TGF-beta1. The pathologic structure was determined by H & E staining and Masson. TGF-beta1, Ras, ERK1/2, and C-Fos levels were determined using western blotting and real-time qPCR. SBP, DBP, and LVMI were reduced significantly in the GJD group compared with the model group. GJD inhibited TGF-beta1, Ras, ERK1/2, and C-Fos expression in LVH. In conclusion, GJD reduced the Ras/ERK1/2 pathway expression, which decreased hypertension-induced heart hypertrophy. GJD may protect hypertension-induced myocardial hypertrophy by altering gene expression patterns in the heart.

## 1. Introduction

Hypertension has the potential to become a major factor in global health because it increases death and disability rates among people in a wide range of countries. Hypertension affects about 35.533 million people in China, and over 75 million American adults (32%) have high blood pressure—one out of every three individuals [1]. Hypertension is associated with an increased risk of organ damage, including cardiac hypertrophy and heart disease, and it remains a major risk factor for illnesses and mortality worldwide despite a significant collection of research showing the advantages of antihypertensive drugs [2]. Increased arterial blood pressure causes organ damage via hemodynamic stress, resulting in hypertensive vasculopathy and left ventricular hypertrophy [3, 4].

Left ventricular hypertrophy (LVH) caused by hypertension, a common type of cardiac remodeling, is an important mechanism for adaptation to a long-term enhanced afterload caused by high blood pressure [5]. Despite its compensating function, this adaptation is responsible for the exacerbation of cardiovascular complications. Around 36% to 41% of all hypertensives have LVH [6], and heart failure pathogenesis is highly impacted by LVH, with 1- year death rates as high as 22% [7]. Early detection and treatment of LVH are critical to achieving the desired blood pressure reduction.

Mitogen-activated protein kinases (MAPKs) have been proposed as significant mediators of the ventricular hypertrophy response in culture cells and genetically modified animals [8]. Many studies have revealed that the Ras/ERK1/2 signaling pathway plays a role in cardiac hypertrophy and human malignancies in both humans and animals [9–13]. Activation of this signal pathway induced left ventricular hypertrophy [14–16]. Therefore, traditional Chinese medicine with antihypertensive effects and regulation of the Ras/ERK1/2 signaling pathway may reverse cardiac cell hypertrophy. The traditional Chinese medicine GJD (patent published number (CN114246896A)) includes six botanical drugs: Uncaria rhynchophylla (Miq.) Miq. Ex Havil. (Gouteng) (Rubiaceae), Salvia miltiorrhiza Bunge (Danshen) (Lamiaceae), Pueraria lobata (Willd.) Ohwi. (Gegen) (Fabaceae), Eucommia ulmoides Oliv. (Duzhong) (Eucommiaceae), *Prunella vulgaris* L. (Xiakucao) (Lamiaceae), and *Achyranthes bidentata* Blume (Niuxi) (Amaranthaceae) were designed to treat hypertension. The herbal medicines in GJD such as Uncaria rhynchophylla (Miq.) Miq. ex Havil has been shown to lower blood pressure, reverse cardiac hypertrophy by inhibiting the MAPK pathway, and reduce the force and frequency of cardiac muscle contraction [17, 18]. In addition, Salvia miltiorrhiza Bunge may protect against cardiomyopathy and improve the cardiac function of diabetic rats by reducing myocardial TGF-beta1 expression [19]. For the treatment of cardiovascular disease, Pueraria lobata (Willd.) Ohwi is commonly utilized in eastern Asia. More than a dozen clinical experiments have demonstrated that puerarin dramatically reduces cardiac hypertrophy and fibrocyte proliferation and activation [20]. Eucommia ulmoides Oliv. has shown significant therapeutic effects on hypertension, and its active constituents have been widely explored for treating cardiovascular problems, including ventricular hypertrophy, myocardial fibrosis, and cardiac remodeling [21]. In addition, *Prunella vulgaris* L. and *Achyranthes bidentata* Blume have cardiovascular therapeutic properties, such as lowering blood pressure [22, 23]. Our work's primary goal is to determine how ERK signaling causes LVH and whether GJD efficiently lowers LVH through the Ras/ERK1/2 pathway.

## 2. Materials and Methods

### 2.1. Materials

GJD: Uncaria rhynchophylla (Miq.) Miq. ex Havil. (Gouteng, Lot No. 20200901); Salvia miltiorrhiza Bunge (Danshen, Lot No. 20191001); Pueraria lobata (Willd.) Ohwi (Gegen, Lot No. 20191001); Eucommia ulmoides Oliv. (Duzhong, Lot No. 20190901); *Prunella vulgaris* L. (Xiakucao, Lot No. 20190701); *Achyranthes bidentata* Blume (Niuxi, Lot No. 20200901) were obtained from Heilongjiang Xiushengtang Pharmaceutical Co., Ltd. Captopril (Meilun Biotechnology Co. Ltd. Dalian); TGF-beta 1 kit (Jiancheng Bioengineering Institute, Nanjing); ALC-NIBP noninvasive BP system (Alcott Biotechnology Co. Ltd. Shanghai); and AL204 electronic balance (Mettler-Toledo Measurement Co. Ltd. Changzhou) were used.

### 2.2. Drug Preparation

GJD botanical drugs are: Uncaria rhynchophylla (Miq.) Miq. ex Havil. (Gouteng), 10 g; Salvia miltiorrhiza Bunge (Danshen), 25 g; Pueraria lobata (Willd.) Ohwi (Gegen) 30 g; Eucommia ulmoides Oliv. (Duzhong) 15 g; *Prunella vulgaris* L. (Xiakucao), 15 g; *Achyranthes bidentata* Blume (Niuxi), 20 g. First, a 60% ethanol solution was mixed with botanical medications (Salvia miltiorrhiza, Pueraria lobata, Eucommia ulmoides, *Prunella* vulgaris, and *Achyranthes bidentata*) at a material-liquid ratio of 1 g: 10 ml, soaked for 30 min, and decocted twice for 1.5 hours (keeping the solution slightly boiling during the reflux process). Six layers of degreasing gauze should be used to filter the extracts. The ethanol was then distilled by using a rotary evaporator, and the extracts were dried under decreased pressure and vacuum to obtain the powder (Salvia miltiorrhiza, Pueraria lobata, Eucommia ulmoides, *Prunella vulgaris*, and *Achyranthes bidentata*). The second step involved incubating Uncaria rhynchophylla for half an hour in a 70% alcohol solution (1 g: 10 ml). Then, we decocted the sample twice at 65∼75°C for 2 hours. Afterward, the Uncaria rhynchophylla was filtered, distilled, and dried in the same manner as stated before to obtain the extract powder. As a final step, GJD was made by grinding together the two powders.

### 2.3. Animal

This study utilized male ten-week-old (body weight 250 ± 15 g) SHR (*n* = 35) and WKY (*n* = 7) rats (Vital River Laboratory Animal Technology Co., Ltd., Beijing) under the experimental animal approval license number: SYXK(Hei)2018-007. The rats were maintained in a standard room temperature environment of 21 ± 4°C, relative humidity 52 ± 5%, and a light 12 h/12 h light-dark cycle and were free to eat and drink. The study protocol was reviewed and approved by the Heilongjiang University of Chinese Medicine's Experimental Animal Centre and Animal Care and Use Committee (approval no: 2020031203).

### 2.4. Groups and Drug Doses

Following a one-week adaptation period, 35 SHR rats were divided into five groups. SHR as a model group (Model), low dosage GJD group (GJD-L) (1.36 g/kg/d), medium dose GJD group (GJD-M) (2.72 g/kg/d), high dose GJD group (GJD-H) (5.44 g/kg/d), and captopril group (CAPT) (13.5 mg/kg/d). The 7 Wistar–Kyoto (WKY) rats in the control group (Control). The same volume of distilled water was gavaged to the rats in the control and model groups. The US Food and Drug Administration's recommended body surface area was used to convert human dosage to animal dosage.

### 2.5. Blood Pressure and Heart Rate Monitoring

Every week, SBP and DBP, as well as heart rate (HR), were monitored with the tail-cuff method using ALC-NIBP a noninvasive blood pressure system. Measurements were taken three times per rat and an average value was reported. All rats were treated for six weeks. At the end of the experiments, sodium pentobarbital, 45 mg/kg, was injected intraperitoneally to anesthetize the rats. Blood samples were obtained from the abdomen artery and promptly centrifuged at 3500 rpm for 10 min to extract serum. The rats' hearts were immediately removed and washed with normal cold saline before being dried on filter paper. The left ventricle's mass was measured using an electronic scale. LVMI was then calculated using (left ventricular weight (mg)/body weight (g)). Left ventricular dissected parts were fixed in 4% paraformaldehyde for hematoxylin and eosin (HE) and Masson staining, and part of the left ventricular was frozen −80°C for Western blot and RT-qPCR.

### 2.6. ELISA Kit of TGF-beta1

The ELISA kit was used to measure TGF-beta1 levels in serum (Serial number: H034-2). The kit was assembled following the manufacturer's instructions.

### 2.7. Histopathological Examination (H & E and Masson Trichrome Staining)

Fresh tissues were removed from the left ventricular and fixed in 4% paraformaldehyde for 24 hours at 4°C, followed by dehydration. After drying and embedding in paraffin, the fixed tissues were sectioned at a thickness of 5 microns. After dewaxing the paraffin sections in xylene solution and treating them with gradient ethanol, they were immersed in distilled water to remove any leftover wax. To stain the slides, they were dipped in the following dye kits and rinsed: H & E dye kit (G1005; Servicebio, China) and Masson dye kit (G1006). Finally, the films were dried, sealed, and checked under an upright microscope (Nikon Japan, Eclipse CI-l).

### 2.8. RT-qPCR

Total RNA was extracted from the left ventricular tissue of three rats chosen randomly using Trizol reagent (Takara). PrimeScript RT Kit (Takara) was used for cDNA synthesis. We used TB Green® Premix Ex Taq™ II (Tli RNaseH Plus) (Takara) in a real-time PCR experiment. Gene expression level was quantified using QuantStudioTM3 Real-time PCR system. The data were calculated and analyzed using the 2^−ΔΔCt^ technique, and GAPDH was used as an internal control to calculate and interpret the data.  Table 1 shows the primers used for PCR amplification.

### 2.9. Western Blot

Total protein was obtained from the left ventricular of three rats selected randomly using RIPA lysis buffer with PMSF and phosphatase inhibitor (Servicebio, China), and protein concentration was measured using a BCA protein detection kit (Servicebio, China). SDS-PAGE was used to separate the whole protein, which was subsequently transferred to 0.45 *μ*M on the PVDF membrane (Servicebio, China). Before incubating with the primary antibody overnight at 4°C, the membrane was sealed for one hour at room temperature with 5% nonfat milk. The membrane was then washed three times with TBST, incubated with a secondary antibody for one hour at room temperature, and the protein was identified using an ECL reagent (servicebio, China). Primary antibodies include: TGF-beta 1 (bsm-33287m, 1 : 1,000, Bioss, China), Ras 1 (GB11411, 1 : 1,000, Servicebio, China), ERK1/2 (GB11560, 1 : 1,000, Servicebio, China), C-Fos (GB114125, 1 : 1,000, Servicebio, China), and *β*-actin (GB15001, 1 : 2,000, Servicebio, China).

### 2.10. Statistical Analysis

All data in this research are reported as mean ± SD. Statistical analysis was performed using GraphPad Prism version 7 for Windows, California, USA. One-way ANOVA with Tukey's multiple comparisons as a post hoc test was used to examine statistical differences. A *P* value of <0.05 was used to indicate statistical significance.

## 3. Results

### 3.1. Effect of GJD on SBP, DBP, HR, and Body Weight

**Figure 1:**
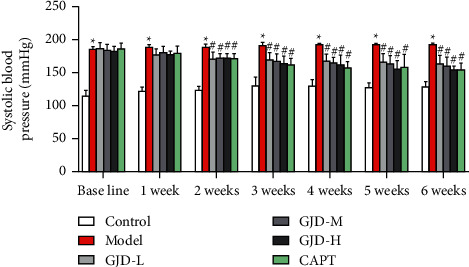
The effect of GJD on blood pressure (SBP and DBP), heart rate, and body weight. (a) SBP, (b) DBP, (c) HR, and (d) body weight. (*n* = 7). Values are means ± SD. Control: WKY (Wistar–Kyoto rats), model: spontaneously hypertensive rats (SHRs), GJD-L: SHR received GJD low dose, GJD-M: SHR received GJD medium dose, GJD-H: SHR received GJD high dose, and CAPT: SHR received captopril. ^*∗*^*P* < 0.05 vs. control and ^#^*P* < 0.05 vs. model.

After six weeks of therapy, in comparison to the model group, SBP and DBP were significantly lower in the GJD-L, GJD-M, GJD-H, and CAPT groups; however, there were no wide variations between the GJD-H and CAPT groups, as shown in Figures 1(a) and 1(b). Furthermore, there were no significant variations in HR and body weight across the groups, as shown in Figures 1(c) and 1(d)).

### 3.2. GJD's Effect on Left Ventricular Histopathology

**Figure 2:**
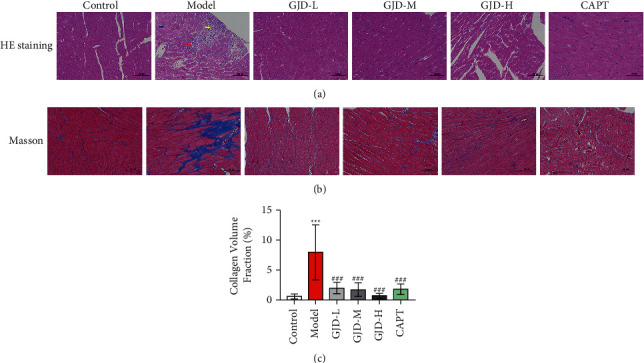
The effect of GJD on morphologic changes and collagen of left ventricular tissue by H & E and Masson staining and collagen volume fraction in each group: (a) H & E staining in each group, (b) Masson staining in each group (fibrotic tissues labeled blue, and ventricular cells marked red), and (c) collagen volume fraction (200x magnification images) (*n* = 7). Control: WKY (Wistar–Kyoto rats), model: spontaneously hypertensive rats (SHRs), GJD-L: SHR received GJD low dose, GJD-M: SHR received GJD medium dose, GJD-H: SHR received GJD high dose, and CAPT: SHR received captopril. ^*∗*^*P* < 0.05, ^*∗∗*^*P* < 0.01, and ^*∗∗∗*^*P* < 0.001 vs. control. ^#^*P* < 0.05, ^##^*P* < 0.01, and ^###^*P* < 0.001 vs. model.

Histological exams were performed six weeks following therapy to assess left ventricular structure. In comparison to the control group, the model group showed a small amount of localized myocardial fiber necrolysis, replaced by hyperplastic connective tissue (yellow arrow) and was accompanied by punctate lymphocytic infiltration (red arrow), and mild watery degeneration with loose cytoplasm and light staining was seen in myocardial fibers (blue arrows), as shown in Figure 2(a). There was no necrosis or fibrosis in the GJD-L, GJD-M, GJD-H, and CAPT groups compared to the model group, as shown in Figure 2(a). Furthermore, we measured collagen concentration in cardiac tissue slices using Masson staining, and consequently, fibrotic tissues were labeled blue, whereas myocardial cells were marked red, as shown in Figure 2(b). The collagen volume fraction (CVF) of all GJD groups was reduced significantly compared to the model group. The GJD-H rats had the most significant reduction in CVF, as shown in Figure 2(c).

### 3.3. GJD's Effect on Left Ventricular Mass Index (LVMI)

**Figure 3:**
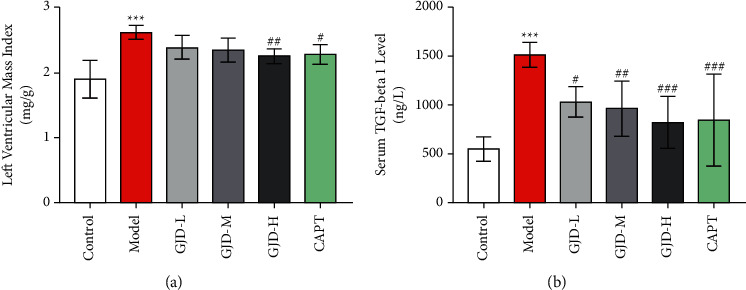
The effect of GJD on left ventricular mass index in each group and serum TGF-beta 1 in each group. (a) Left ventricular mass index. (b) Serum TGF-beta 1 (*n* = 7). Values are means ± SD. Control: WKY (Wistar–Kyoto rats), model: spontaneously hypertensive rats (SHRs), GJD-L: SHR received GJD low dose, GJD-M: SHR received GJD medium dose, GJD-H: SHR received GJD high dose, and CAPT: SHR received captopril. ^*∗*^*P* < 0.05, ^*∗∗*^*P* < 0.01, and ^*∗∗∗*^*P* < 0.001 vs. control. ^#^*P* < 0.05, ^##^*P* < 0.01, and ^###^*P* < 0.001 vs. model.

After 6 weeks of therapy, LVMI decreased significantly in GJD-H and CAPT groups compared with the model group; however, there was no significant reduction in LVMI in the GJD-L low and GJD-M groups, as shown in Figure 3(a).

### 3.4. TGF-beta1 ELISA Kit in Serum

After six weeks of gavage administration, TGF-beta1 levels were identified in serum collected from the abdominal aorta. The TGF-beta1 levels were significantly higher in the GJD-L, GJD-M, GJD-H, and CAPT groups than in the model group. The SHRs in the GJD-H and CAPT groups had the highest significant decrease in TGF-beta1 levels, as shown in Figure 3(b).

### 3.5. Real-Time qPCR Analysis of GJD's Effect on mRNA of Ras/ERK1/2 Signaling

**Figure 4:**
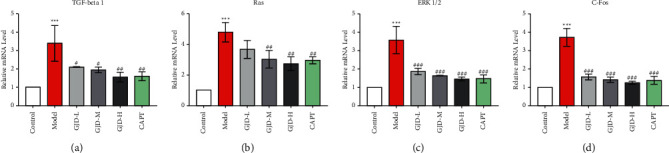
GJD's effect on Ras/ERK1/2 signaling mRNA expression in the left ventricular tissue. (a) mRNA expression of TGF-beta 1, (b) mRNA expression of Ras, (c) mRNA expression of ERK1/2, and (d) mRNA expression of C-Fos (*n* = 3). Values are means ± SD. Control: WKY (Wistar–Kyoto rats), model: spontaneously hypertensive rats (SHRs), GJD-L: SHR received GJD low dose, GJD-M: SHR received GJD medium dose, GJD-H: SHR received GJD high dose, and CAPT: SHR received captopril. ^*∗*^*P* < 0.05, ^*∗∗*^*P* < 0.01, and ^*∗∗∗*^*P* < 0.001 vs. control. ^#^*P* < 0.05, ^##^*P* < 0.01, and ^###^*P* < 0.001 vs. model.

TGF-beta1, Ras, Erk1/2, and C-Fos mRNA expressions in the left ventricular tissue were also measured using real-time qPCR. TGF-beta1 mRNA levels in the model were significantly greater than in the control group. However, TGF-beta1 mRNA levels were significantly lower in the GJD-L, GJD-M, and GJD-H groups following treatment compared with the model group. The rats in the GJD-H and CAPT groups showed the greatest reduction in TGF-beta1 levels, as shown in Figure 4(a). The Ras levels were significantly lower in the GJD-M, GJD-H, and CAPT groups than in the model group, although not in the GJD-L group, as shown in Figure 4(b). The mRNA level of ERK1/2 in the GJD-L, GJD-M, GJD-H, and CAPT groups was significantly lower than in the model group, and there was no statistically significant difference across the groups, as shown in Figure 4(c). C-Fos mRNA levels were reduced significantly in all GJD groups compared with the model group, as shown in Figure 4(d).

### 3.6. WB Analysis of GJD's Effect on Ras/ERK1/2 Pathway Protein Expression

**Figure 5:**
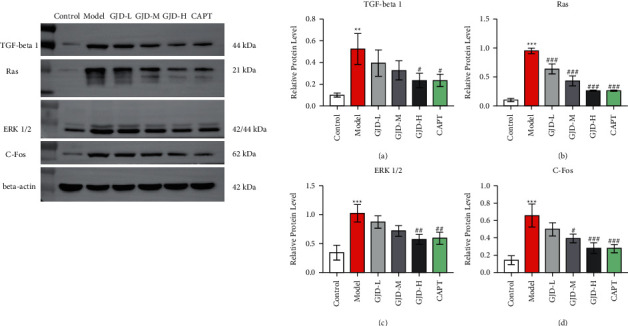
GJD's effect on the protein expression of Ras/ERK1/2 pathway of TGF-beta 1, Ras, ERK1/2, and C-Fos in left ventricular tissue in each group. (a) Expression level of TGF-beta1, Ras, ERK1/2, and C-Fos, (b) expression level of TGF-beta 1, (c) expression level of Ras, (d) expression level of ERK1/2, and (e) expression level of C-Fos (*n* = 3). Values are means ± SD. Control: WKY (Wistar–Kyoto rats), model: spontaneously hypertensive rats (SHRs), GJD-L: SHR received GJD low dose, GJD-M: SHR received GJD medium dose, GJD-H: SHR received GJD high dose, and CAPT: SHR received captopril. ^*∗*^*P* < 0.05, ^*∗∗*^*P* < 0.01, and ^*∗∗∗*^*P* < 0.001 vs. control. ^#^*P* < 0.05, ^##^*P* < 0.01, and ^###^*P* < 0.001 vs. model.

Western blotting was used to evaluate the protein expression of the Ras/ERK1/2 pathway, which includes TGF-beta 1, Ras, ERK1/2, and C-Fos.  Figure 5(a) demonstrates that the TGF-beta1 level in the model group was significantly higher than in the control group. TGF-beta1 levels were significantly lower in the GJD-H and CAPT groups than in the model group. However, the decrease in the GJD-L and GJD-M groups was not statistically significant. As shown in Figure 5(b), the Ras level was significantly elevated in the model group compared to the control group; however, when compared to the model group, the Ras level was significantly decreased in the GJD-L, GJD-M, GJD-H, and CAPT groups. As shown in Figure 5(c), ERK1/2 levels were significantly lower in the GJD-H and CAPT groups compared to the model group. However, the decrease in the GJD-L and GJD-M groups was not statistically significant. As shown in Figure 5(d), C-Fos levels were significantly lower in the GJD-M, GJD-H, and CAPT groups compared to the model group, but the decrease in the GJD-L group was not statistically significant. There is no significant difference between the GJD-H and CAPT groups, as shown in Figure 5(d).

## 4. Discussion

GJD consists of 6 botanical drugs, Uncaria rhynchophylla (Miq.) Miq., Salvia miltiorrhiza Bunge, Pueraria lobata (Willd.) Ohwi, Eucommia ulmoides Oliv., *Prunella vulgaris* L, and *Achyranthes bidentata* Blume. Here, an in vivo examination of the effect of GJD on hypertensive LVH treatment was investigated. All SHRs showed elevated serum TGF-beta1 levels, LVMI, and collagen fibers in cardiac tissue. Moreover, western blot and RT-qPCR detected elevated expression of TGF-beta1, Ras, ERK 1/2, and C-Fos in left ventricular tissue. GJD effectively reduced blood pressure, serum TGF-beta1 levels, and LVMI and thus inhibited myocardial fibrosis. In addition, GJD reduced the protein expression of TGF-beta1, Ras, ERK 1/2, and C-Fos in left ventricular tissue. Taken together, GJD reduced the TGF-beta1, Ras, ERK1/2, and C-Fos pathway expression, which decreased hypertension-induced left ventricular hypertrophy.

SHRs may experience a rise in blood pressure and an increase in peripheral vascular resistance during four to six weeks without medication or surgery, which accurately reflects the pathological process of humans with primary hypertension [24, 25]. A higher prevalence of LVH was also observed in SHR rats than in WKY rats in other studies, as was the case in earlier investigations using the SHR model [26, 27]. Consequently, SHRs were selected as the study's animal model.

Left ventricular hypertrophy (LVH) is a compensatory response to uncontrolled hypertension as well as an indicator of hypertensive target organ damage, and it is also a risk factor for cardiovascular morbidity and mortality, such as heart failure, arrhythmias, stroke, and sudden cardiac death [9, 28]. Prevention of the development of LVH and target organ damage should be, therefore, an important therapeutic goal for the management of arterial hypertension [29–31]. The most controllable parameter for achieving this objective is adequate blood pressure (BP) management with suitable antihypertensive medicines [31, 32]. In our study, GJD results revealed a significant decrease in systolic and diastolic blood pressure at varying concentrations, indicating that GJD might be utilized to reduce BP and is effective for hypertension therapy. As such, we hypothesized that GJD might contribute to a delay in the development of LVH in our SHRs.

High blood pressure has been proven in chronic hypertension to cause permanent thickening of the wall and alterations concerning particular situations such as LVH and heart failure [33]. Left ventricular hypertrophy (LVH) is a common feature of hypertension-related heart disease characterized by cardiomyocyte enlargement and fibrosis caused by cardiac fibroblast proliferation [34]. Long-term pressure overload causes increased depositing of cardiac collagen fibers, increased collagen concentration, imbalanced collagen ratio, and disorderly arrangement, resulting in alterations in heart function and structure and, consequently, a rise in the risk of cardiovascular events. LVH is a major indicator for the evaluation of myocardial hypertrophy, including pathological manifestations such as the elevation of LVMI or cardiomyocyte hypertrophy, as well as the aggravation of myocardial interstitial fibrosis, which could be further increased in response to the continuous enhancement of pressure overload [35–38]. In the current research, we also found that SHRs had significant increases in LVMI and CVF, an indicator reflecting the fibrosis of the myocardium, which was also augmented, but this was reversed by GJD treatment.

TGF-beta 1 is linked to extracellular matrix deposition and has been identified as a target for the therapy of organ fibrosis [39]. TGF-beta 1 has been linked to the development of cardiac hypertrophy and heart failure in the cardiovascular system, as well as an increase in both cardiomyocyte growth and intercellular fibrosis [40]. Pressure overload, cardiac infarction, immunological damage, and other stimuli may activate the TGF-beta 1 signal and begin fibrosis, resulting in a substantial amount of collagen deposition [41]. Clinical investigations have demonstrated that a rise in plasma TGF-beta 1 levels in hypertensive individuals is directly associated with hypertension and its target organ damage [42]. Under pressure overload, hypoxia-inducible factor 1 (HIF-1) protects the heart and aorta of mice by suppressing the expression of TGF-beta 1-Smad2/3 and TGF-beta 1-ERKl/2 [43]. Our results show that GJD inhibited the expression of TGF-beta1/Ras/Erk1/2/C-Fos as detected by Western blotting and RT-qPCR. Certain studies have reported a positive correlation between TGF-beta1 and the ERK signaling pathway [44, 45]. As documented in noncanonical signaling of TGF-beta1, TGF-beta-RIi phosphorylates and activates Ras, which further activates the ras/raf/MEK signaling cascade to convey signals from their receptors to control gene expression [12, 14, 46, 47]. Over-expression of activated Ras in the heart results in cardiac hypertrophy and stimulates the kinase pathway ERK [48, 49]. Extracellular signal-regulated kinases (ERK)1/2, a subunit of MAPK, are required to regulate gene expression in cardiac hypertrophy [46, 47]. The phosphorylation of ERK1/2 has been linked to an increase in the expression of the cardiac transcription factor c-Fos [12, 50]. This suggests that c-Fos plays a major role in cardiac hypertrophy [51, 52]. The inhibition of ERK could improve the LVW/BW ratio and systolic blood pressure to exert anti-hypertrophic effects [53].

Many of GJD's active components reported in the patent may inhibit TGF-beta 1 and Ras/Erk1/2/C-Fos mRNA/protein levels. Among others, Isorhynchophylline is one of the major active components in Uncaria rhynchophylla. Isorhynchophylline can inhibit the phosphorylation of the MAPK signaling pathway in AngII-treated ventricular myocytes, which suggests that Isorhynchophylline has an inhibitory effect on cardiac hypertrophy via the MAPK signaling pathway [17]. Rhynchophylline and Isorhynchophylline may decrease vascular adventitial fibroblast proliferation by reducing c-Fos and TGF-beta1 mRNA expression in spontaneously hypertensive rats [54]. Tanshinone IIA, the main ingredient in Salvia miltiorrhiza Bunge, blocks vascular smooth muscle cell proliferation by inhibiting the ERK1/2 signal transduction pathway and downregulating c-FOS expression [55] and plays an estrogenic protective effect on vascular endothelial cells by downregulating ERK1/2 expression [56]. Puerarin, the active ingredient in Pueraria lobata, can prevent isoprenaline-induced cardiac fibrosis in rats. Its mechanism may involve reducing transforming growth factor-1 expression [57]. In our study, the SHR in the model group had LVH and overexpression of the TGF-beta1/Ras/Erk1/2/C-Fos signaling pathway, but GJD treatment inhibited the ventricular hypertrophy, decreased the level of TGF-beta1, Ras, Erk1/2, and C-Fos, possibly due to the active constituents in GJD botanical medications. This may show how ERK signaling promotes LVH, and GJD decreases it via Ras/ERK1/2.

In GJD treating LVH through TGF-beta 1, Ras, Erk1/2, and C-Fos, it is unclear how many of these chemical compounds possess anti-hypertrophic bioactivity. We also do not know whether these active components act individually, additively, or synergistically. As a result, several questions must be resolved in our future investigations before GJD may be developed as an anti-hypertension/anti-hypertrophic medication. Future studies must use RNA-seq to discover GJD molecular pathways and LVH biomarkers, and this innovative technology could elucidate GJD's LVH therapeutic processes.

## 5. Conclusion

In conclusion, GJD lowered blood pressure and improved left ventricular hypertrophy in SHRs via regulating the Ras/ERK1/2 pathway, which may provide a theoretical basis to assist further studies in hypertensive myocardial hypertrophy research.

## Figures and Tables

**Table 1:** Primer sequences.

Gene	Forward primer (5′–3′),	Reverse primer (5′–3′),
TGF-beta1	CTGCTGACCCCCACTGATAC	AGCCCTGTATTCCGTCTCCT
Ras	CCATCAGTACAGGGAGCAGA	CGGGTCTTGGCTGATGTTTC
ERK1/2	TCAAGCCTTCCAACCTC	GCAGCCCACAGACCAAA
C-Fos	ACGGAGAATCCGAAGGGAAAGGAA	TCTGCAACGCAGACTTCTCGTCTT
GAPDH	TGCACCACCAACTGCTTAG	GATGCAGGGATGATGTTC

## Data Availability

The data used to support the findings of this study are available from the corresponding author upon request.
